# Outcomes of kidney transplantation in patients with congenital anomalies of the kidney and urinary tract: a propensity-score-matched analysis with case-control design

**DOI:** 10.55730/1300-0144.5613

**Published:** 2023-02-01

**Authors:** Özgür Akın OTO, Şafak MİRİOĞLU, Halil YAZICI, Ahmet Burak DİRİM, Nurane GÜLLER, Seda ŞAFAK, Erol DEMİR, Ayşe Serra ARTAN, Yasemin ÖZLÜK, Aydın TÜRKMEN, Yaşar ÇALIŞKAN, Krista L. LENTINE

**Affiliations:** 1 Division of Nephrology, İstanbul School of Medicine, İstanbul University, İstanbul, Turkey; 2 Division of Nephrology, School of Medicine, Bezmialem Vakif University, İstanbul, Turkey; 3 Department of Pathology, İstanbul School of Medicine, İstanbul University, İstanbul, Turkey; 4 Division of Nephrology and Hypertension, School of Medicine, Saint Louis University, Saint Louis, USA

**Keywords:** Congenital abnormalities of kidney and urinary tract, graft loss, outcomes, transplantation

## Abstract

**Background/aim:**

We compared long-term outcomes after kidney transplantation (KTx) in patients with and without congenital anomalies of the kidney and urinary tract (CAKUT).

**Materials and methods:**

KTx recipients (KTRs) with CAKUT in 1980–2016 were identified; their hard copy and electronic medical records were reviewed and compared to a propensity-score-matched control group (non-CAKUT) from the same period. The primary outcomes were graft loss or death with a functioning graft; secondary outcomes included posttransplant urinary tract infections (UTIs) and biopsy-proven rejection (BPR).

**Results:**

We identified 169 KTRs with CAKUT and 169 matched controls. Median follow-up was 132 (IQR: 75.0–170.0) months. UTIs were more common in CAKUT patients compared to non-CAKUT group (20.7% vs 10.7%; p = 0.01). Rates of BPR were similar between the two groups. In Kaplan–Meier analysis, 10-year graft survival rates were significantly higher in the CAKUT group than in the non-CAKUT group (87.6% vs 69.2%; p < 0.001), while patient survival rates were similar. In multivariate Cox regression analyses, CAKUT (HR: 0.469; 95% CI: 0.320–0.687; p < 0.001) and PRA positivity before transplantation (HR: 3.756; 95% CI: 1.507–9.364; p = 0.005) predicted graft loss.

**Conclusions:**

Graft survival in KTRs with CAKUT appears superior to KTRs without CAKUT. Transplant centers should develop multidisciplinary educational and social working groups to support and encourage CAKUT patients with kidney failure to seek for transplants.

## 1. Introduction

Congenital anomalies of the kidney and urinary tract (CAKUT), the most common cause of end-stage kidney disease (ESKD) in children, encompasses a large spectrum of conditions including renal agenesis or hypodysplasia, ureteropelvic junction obstruction, multicystic dysplastic kidney, congenital megaureter, ureterovesical junction obstruction, pelvis, ureter and/or kidney duplication, posterior urethral valves (PUVs), and vesicoureteral reflux (VUR) [1, 2]. Although CAKUT is known to account for approximately 50% of ESKD in children, it accounts for less than 5% of ESKD in adult patients [3].

The prognosis of patients who receive kidney transplantation (KTx) for CAKUT may differ compared to those with non-CAKUT causes. Previous studies have suggested that patients with CAKUT may have better prognosis in the posttransplant period [4, 5]. On the other hand, in several forms of CAKUT associated with bladder dysfunction, a predisposition to posttransplant pyelonephritis and/or obstructive nephropathy may develop, which may have adverse effects on graft survival. Although some studies have shown that the risk of graft loss does not increase in patients suffering from PUV with frequent bladder dysfunction [6], other studies have found higher mean serum creatinine level, increased rate of urological complications, and higher rates of urinary tract infections (UTIs) after KTx [6–8]. In another study comparing posttransplant outcomes of patients with VUR and controls, there were no significant differences in posttransplant complications and patient and graft survival rates despite the higher incidence of UTIs in VUR patients [9]. Given these conflicting results, the posttransplant course of patients with CAKUT needs further evaluation.

To advance understanding of the implications of CAKUT in the context of KTx, we conducted a matched controlled study to compare the long-term outcomes after KTx in transplant recipients with CAKUT and non-CAKUT etiologies of kidney failure.

## 2. Patients and methods

### 2.1. Patient and control selection

In this case-control study, from our database, we identified 169 KTx recipients (KTRs) with CAKUT who underwent KTx between 1980 and 2016 at Istanbul School of Medicine. The inclusion and exclusion criteria of the study are listed as follows: inclusion criteria: CAKUT proven by imaging and other diagnostic methods, being over 18 years of age at enrollment; and exclusion criteria: autosomal dominant polycystic kidney disease, autosomal dominant tubulointerstitial cystic disease, patients without informed consent, lack of follow-up data.

**Figure 1:**
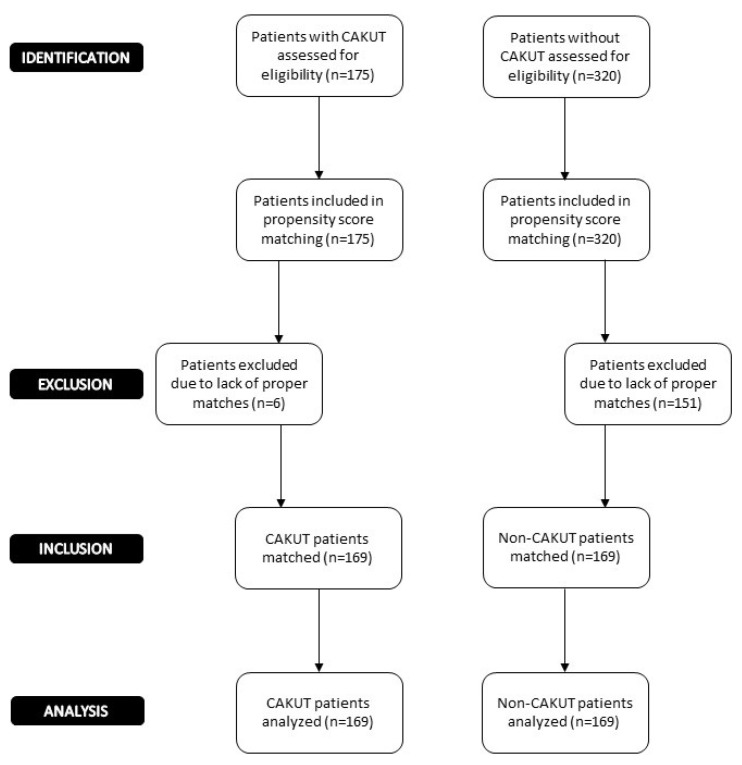
Study flow chart (CAKUT: congenital anomalies of the kidney and urinary tract).

Control patients were from a database of 320 KTRs whose etiologies of kidney failure were other than CAKUT (non-CAKUT) (Figure 1). In the control group, primary kidney disease was glomerulonephritis (GN) in 121 patients (71.6%). Of these 121 patients, 58 had focal segmental glomerulosclerosis (FSGS), 33 had IgA nephropathy (IgAN), 21 had membranoproliferative glomerulonephritis (MPGN), and 9 had membranous nephropathy (MN). Primary kidney disease was unknown in the remaining 48 patients (28.4%). In patients with unknown primary kidney disease, using ultrasonography and voiding cystourethrogram, CAKUT was ruled out during pretransplant evaluation.

Patient follow-up was initiated at the time of transplant and continued until the primary study outcomes including graft loss defined as need for dialysis or retransplantation, or death with a functioning graft. During baseline evaluation, which is the time of transplant, all study patients have kidney failure (eGFR less than 15 mL/min/1.73 m^2^). To assemble matched controls, propensity scores were calculated using a multivariable logistic regression model based on potentially confounding differences including recipient sex, age, donor type (living or deceased), donor sex, number of HLA mismatches, having a preemptive transplant, time spent on dialysis before transplantation, and duration of posttransplant follow-up between two study groups. In order to secure the most similar transplant recipient without CAKUT for each recipient with CAKUT, a matching process using the nearest neighboring method in 1:1 ratio was performed [10].

Baseline and follow-up clinical information were collected from our medical records which included both hard copy and electronic files. Investigator team collected and reviewed all available data from the records.

### 2.2. Covariates and other exposures

Patients were evaluated in our transplantation outpatient clinic immediately after discharge. Weekly and biweekly appointments were held in the first three months. Patients with stable graft function were evaluated every month during the first year, and every three months thereafter. Before 1988, all patients used azathioprine (AZA) and low- to intermediate-dose prednisolone (7.5–10 mg/day) as maintenance. Calcineurin inhibitors (CNIs) were added to the regimen in 1988, starting with cyclosporine (CsA) [11]. Immunosuppressive maintenance therapy consisted of a CNI [CsA or tacrolimus (Tac)], an antimetabolite [mycophenolic acid (MPA) or AZA] and low-dose prednisolone (5 mg/day). Target serum trough levels of CsA and Tac after transplantation were 200–300 ng/mL and 8–12 ng/mL for the first 3 months, and 50–150 ng/mL and 4–8 ng/mL for subsequent months, respectively. AZA and mycophenolate mofetil (MMF) doses were 1.5 mg/kg per day and 2 g/day (1440 mg/day for mycophenolate sodium), respectively. Induction therapy with antithymocyte globulin (ATG) was used for transplantations from living donors with high immunological risk and deceased donors after 1990. Starting from 2002, an interleukin-2 blocker (basiliximab, 20 mg on day 0 and 4) have been used for transplantations from living donors without high immunological risk. Alterations were made in treatment strategies according to immunologic risk and posttransplant complications.

All patients received *Pneumocystis jirovecii* prophylaxis with trimethoprim/sulfamethoxazole in the first year after transplantation. UTI was defined as the presence of ≥10^5^ colony-forming units (CFU)/mL of bacteria in a patient with pyuria and clinical symptoms and signs of infection. Asymptomatic bacteriuria was excluded. Two or more episodes of UTI in consecutive six months in any time during the follow-up were defined as recurrent UTIs [12]. Estimated glomerular filtration rates (eGFRs) of patients were calculated by using Chronic Kidney Disease Epidemiology Collaboration (CKD-EPI) formula [13]. Urinary protein-to-creatinine ratio (uPCR) in the first-morning urine specimen was used to measure level of proteinuria.

Biopsies were performed only with indication, which included an unexplained increase in serum creatinine or new-onset proteinuria (≥1 g/g). Banff diagnostic categories and related criteria at the time of each allograft biopsy were used for the final pathological diagnosis [14–18]. Posttransplant complications, deaths, and causes of graft loss were recorded. Adherence problem was determined with the notation of healthcare personnel.

### 2.3. Study outcomes

The primary outcomes were graft loss defined as need for dialysis or retransplantation, or death with a functioning graft. Biopsy-proven rejection (BPR) and recurrent posttransplant UTIs were the secondary outcomes. Medication adherence was assessed at follow-up visits and defined as any missed doses; having missed at least one dose of medication and/or having missed two or more consecutive doses over the past 4 weeks.

### 2.4. Statistical analyses

Results are reported as the mean ± SD when normally distributed or as the median (interquartile range [IQR]). Study groups were compared with the *t* test or the Mann–Whitney *U* test where appropriate. Differences in the proportions of different patient groups were compared by chi-squared or Fisher’s exact test. Graft survival was analyzed using Kaplan–Meier curves and the survival time for each patient was computed from time of transplant to the last follow-up or the primary outcome. Unbalanced variables remained from propensity score matching and present from the beginning of the study period (recipient age, recipient sex, and duration of dialysis), history of previous KTx, positive (≥30%) panel reactive antibodies (PRA) before transplantation, as well as having CAKUT as primary kidney disease were analyzed using multivariable Cox regression models, which were used to identify graft loss and the associated risk in terms of hazard ratio (HR) and 95% confidence intervals (CI). Statistical analyses were performed using SPSS for Windows (SPSS version 25.0, IBM Corp., Armonk, NY, USA). Kaplan–Meier curves were generated with MedCalc for Windows (MedCalc version 19.0, MedCalc Software, Ostend, Belgium). A p-value of 0.05 or less was considered statistically significant. Our study complied with the Declaration of Helsinki [19], as well as the Declaration of Istanbul 2008, and was approved by the local ethical committee in our institution. The results were presented in line with the STROBE guidelines [20].

## 3. Results

### 3.1. Demographic and clinical features

In total, 338 KTRs (201, 59.4% males) who were followed up for a median of 132 (IQR: 75–170) months were included in the study (Figure 1). The majority of the CAKUT group consisted of VUR (129, 76%). Other diagnoses were as follows: Neurogenic bladder (9, 22.5%), posterior urethral valve (1, 2.5%), urinary tract dilation or anomalies of the ureters (13, 32.5%), renal ectopy/fusion or renal hypoplasia/dysplasia, (10, 25%), and forms of CAKUT that were not otherwise specified (7, 17.5%). After propensity matching, the CAKUT and non-CAKUT groups were similar in terms of donor age, donor sex, recipient sex, donor type, follow-up duration, presence of pretransplant PRA, number of HLA mismatches, proportion of preemptive transplants and immunosuppressive regimens (Table 1). However, there were some residual imbalances in matching: median age of KTRs was lower in the CAKUT group compared to the non-CAKUT group [25.5 (IQR: 20–30) vs 30 years (IQR: 25–38), respectively; p = 0.001]. Time spent on dialysis was longer in the CAKUT group than in the non-CAKUT group [15 (IQR: 5–30) vs 19 (IQR: 6.5–43) months, respectively; p < 0.001]. The number of patients who had previous KTx or PRA positivity at baseline was quite low. Baseline characteristics of all non-CAKUT patients before matched and unmatched patients with non-CAKUT and CAKUT are shown in Tables S1 and S2, respectively.

### 3.2. Study outcomes

A total of 124 patients [CAKUT group (40, 23.7%) and non-CAKUT group (84, 49.7%)] lost their grafts after a median follow-up of 132 (IQR: 75–170) months posttransplant. Main causes of graft loss were BPR (19, 11.2%) and chronic allograft nephropathy (12, 7.1%) in the CAKUT group, while recurrent or de novo GN (36, 21.4%) and BPR (26, 15.5%) caused graft loss in the non-CAKUT group (Table 2). In 76 patients of the non-CAKUT group (45%), recurrent or de novo GN was diagnosed in the follow-up period. Of these 76 patients, 40 had FSGS, 16 had IgAN, 13 had MPGN, 4 had MN, 2 had atypical hemolytic uremic syndrome, and 1 had C1q nephropathy. Death was less common in recipients with CAKUT compared with the non-CAKUT group [9 (5.3%) vs 19 (11.2%); p = 0.001)]. At the end of follow-up, median eGFR of the CAKUT group [53.7 (IQR: 23.9–77.2) mL/min/1.73 m^2^] was significantly higher than that of the non-CAKUT group [32.3 (IQR: 11–67.3) mL/min/1.73m^2^] (p < 0.001). At last follow-up, median proteinuria levels were significantly higher in the non-CAKUT group compared to CAKUT group [1.3 (IQR: 0.1–3) g/g and 0.33 (IQR: 0.1–1.2) g/g, respectively; p < 0.001], as well. Median proteinuria levels at the last visit of patients with unknown etiology in the non-CAKUT group was 0.2 g/day (IQR: 0.1–3.2 g/day), there were no differences in terms of proteinuria between the CAKUT group and patient with unknown etiology in the non-CAKUT group. There were also no differences in terms of proteinuria between the patients in the CAKUT group and patients with unknown etiology in the non-CAKUT group (p = 0.605). However, the number of patients in the group with unknown etiology was very small compared to the GN group (6 vs 163, respectively). Therefore, a separate analysis was not performed.

UTIs and medication nonadherence were more common in patients in the CAKUT group as compared to patients in the non-CAKUT group (20.7% vs 10.7%, p = 0.01 and 9.5% vs 3%, p = 0.01, respectively). Number of patients with BPR, posttransplant PRA and donor specific antibody (DSA) development, BK nephropathy, biopsy-confirmed CNI toxicity, and chronic allograft nephropathy were similar between groups.

**Figure 2:**
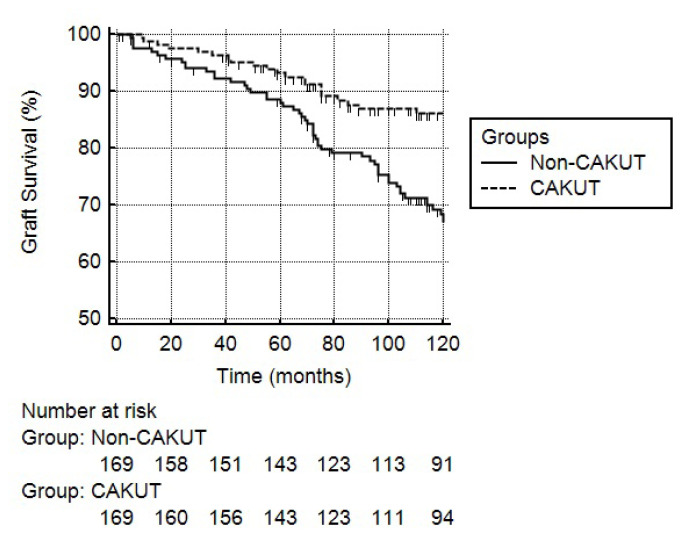
Kaplan–Meier analysis of 10-year graft survival in study groups (p < 0.001 with logrank test).

Five-year Kaplan–Meier survival analysis showed similar graft survival between the groups (p = 0.09), whereas 10-year analysis revealed that graft survival rates were significantly higher in the CAKUT group compared to the non-CAKUT group (87.6% vs 69.2%, respectively, p < 0.001) (Figure 2). However, patient survival was similar between the groups according to 5- and 10-year Kaplan–Meier analyses (p = 0.93 and p = 0.36, respectively).

In multivariate Cox regression analyses, CAKUT as primary kidney disease (HR: 0.469; 95% CI: 0.320–0.687; p < 0.001) and PRA positivity before transplantation (HR: 3.756; 95% CI: 1.507–9.364; p = 0.005) were the predictors of graft loss. Recipient age (HR: 1.009, 95% CI: 0.992–1.027; p = 0.29), recipient sex (HR: 1.071; 95% CI: 0.726–1.580; p = 0.73), and duration of dialysis (HR 0.998, 95% CI 0.994–1.002, p = 0.30) did not predict the graft loss.

In multivariate analyses including PRA, recipient age, recipient sex, and pretransplant dialysis duration to determine the factors predicting graft loss in the CAKUT group, none of these factors were found to be predictive. (For PRA, p = 0.908; HR: 0.869; 95% CI: 0.078–9.676; for recipient age, p = 0.922; HR: 0.998; 95% CI: 0.966–1.032; for recipient sex, p = 0.125; HR: 0.594; 95% CI: 0.305–1.156; for dialysis duration, p = 0.832; HR: 0.999; 95% CI: 0.992–1.006)

### 3.3. Outcomes of patients with VUR nephropathy as primary kidney disease

When patients with primary kidney disease of VUR nephropathy (n = 129) was compared with other patients in the CAKUT group, recipients with VUR nephropathy had a higher frequency of UTIs compared to other recipients with CAKUT (24.8% vs 7.5%, respectively, p = 0.02). However, presence of pretransplant PRA, rates of posttransplant PRA/DSA development, BPR, medication adherence problem, posttransplant VUR, biopsy-confirmed BK nephropathy, CNI toxicity, chronic allograft nephropathy, graft loss, and death did not differ between VUR and other CAKUT patients. At the end of the follow-up, the median eGFR of the VUR group [57.4 (IQR: 30.0–85.3) mL/min/1.73 m^2^] was significantly lower than that of the other patients with CAKUT [80.2 (IQR: 38.9–101.3) mL/min/1.73 m^2^] (p = 0.02). Details of clinical features and outcomes of patients with VUR nephropathy in comparison with other patients with CAKUT are shown in Table 3.

## 4. Discussion

In this retrospective study using propensity score matching, we demonstrated that recipients with CAKUT as primary kidney disease had lower graft loss rates compared to recipients without CAKUT. Although there was no significant difference in rejection rates and patient survival between the study and control groups, higher eGFR and lower proteinuria levels during posttransplant follow-up were found in the CAKUT group.

Various transplantation centers have published the patient and graft survival results of patients with CAKUT and non-CAKUT etiologies of primary kidney disease; however, most of these centers perform pediatric transplantation, and there are only a few studies in the literature examining adult recipients with well-matched control groups [5, 21–23]. Our group previously reported posttransplant prognosis of patients with VUR [9]; however, to the best of our knowledge, there have been no reports comparing adult KTRs with CAKUT against recipients with non-CAKUT etiologies using propensity score matching to control for other predictors of transplant outcome. In a recent study, Monteverde et al. compared the long-term results of CAKUT and non-CAKUT KTRs [24]. In line with our findings, they found that recipients with CAKUT had better last follow-up eGFRs but had higher incidences of UTIs during posttransplant follow-up. The authors did not find any differences regarding 1-, 5-, and 10-year patient survival rates between the groups; however, graft survival was better in patients with CAKUT than in those with non-CAKUT [24]. Another group also showed that non-CAKUT KTRs had lower complication rates and better allograft function [25]. On the other hand, a few studies demonstrated that there were no significant differences regarding graft function between patients with CAKUT and non-CAKUT etiologies [21–24]. The main difference of our study from the studies mentioned above is the composition and selection of the control group. Our study was conducted with an adult patient population and a control group formed by propensity score matching based on potentially confounding differences was used [26].

KTRs are at increased risk of UTIs due to multiple factors such as immunosuppression, double-J stent use, and other manipulations of the urinary tract. Recurrent UTIs during posttransplant period has negative effects on graft functions in the long term due to scarring and tubulointerstitial injury [27]. Although the incidence of UTI was significantly higher in the CAKUT group, a negative outcome for graft survival was not observed in our cohort. Our findings are inconsistent with a recently published study which showed a significant risk for graft loss in patients with UTIs after transplantation [21]. This difference may be related to our routine administration of long-term (1 year) trimethoprim-sulfamethoxazole prophylaxis to all recipients after KTx.

In our cohort, the most common type of CAKUT was VUR nephropathy (76%). Although patients with primary kidney disease of VUR nephropathy had a lower eGFR and a higher incidence of UTIs than other patients with CAKUT, two subgroups were similar in terms of study outcomes, supporting the previous studies [28, 29]. Most of the patients in the control group formed by propensity score matching happened to have GN as primary kidney disease. This situation can be explained by the fact that patients with CAKUT were younger and our university hospital is one of the most important glomerulonephritis referral centers.

Our study has some limitations. It was conducted as a retrospective analysis, and there may be additional sources of confounders not captured in the analyses. Second, we included patients who underwent transplantation as early as 1980. Transplantation field have dramatically changed over these decades. Therefore, a potential era effect should be taken into consideration when interpreting the results. Third, non-CAKUT group consisted of mainly patients with GN, and recurrent or de novo disease was an important cause of graft loss in this group [30], which may have caused an overestimation of the difference in graft loss between study groups. Our study has significant strengths, including use of propensity score matching to help balance potential confounders in assembling the control group. Also, our data represents a follow-up over a decade including a detailed examination of posttransplant complications, and presence of posttransplant PRA and DSA measurements.

The strengths of the study, as well as its superiority over the existing literature, are its multicenter design, which includes only Turkish population data, the use of propensity cross-match that minimizes confounding factors for the control group, long follow-up period (over >10 years), and detailed immunological and infective complication records.

In conclusion, graft survival in KTRs with CAKUT was better than in KTRs with non-CAKUT etiologies and this effect was maintained over a 10-year of follow-up period. Transplant centers should develop multidisciplinary educational and social working groups to support and encourage CAKUT patients with kidney failure to seek for transplants.

## Supplementary material

**Table S1:** Baseline demographic, clinical, and laboratory characteristics of all non-CAKUT patients included in the database used for propensity score matching.

Characteristics	Non-CAKUT (n = 320)
Donor age (years), median (IQR)	46.0 (35.0–54.0)
Donor sex, %	
Male	50.9
Female	49.1
Recipient sex, %	
Male	61.2
Female	38.8
Recipient age (years), median (IQR)	32.0 (25.0–41.0)
Donor type, %	
Living	74.7
Deceased	25.3
Follow-up duration (months), median (IQR)	146.0 (100.0–185.5)
HLA mismatches, %	
≤3	18.1
>3	81.9
Duration of dialysis (months), median (IQR)	18.0 (6.0–36.5)
Pretransplant KRT, %	
No (Preemptive)	12.5
Yes (HD and/or PD)	87.5
Immunosuppressive regimen, %	
Tac/MPA/steroids	51.3
CsA/MPA/steroids	21.9
Tac/AZA/steroids	9.1
CsA/AZA/steroids	3.0
Others	14.7

**Abbreviations:** AZA: azathioprine; CAKUT: congenital anomalies of the kidney and urinary tract; CsA: cyclosporine; HD: hemodialysis; IQR: interquartile range: human leukocyte antigen; KRT: kidney replacement therapy; MPA: mycophenolic acid; PD: peritoneal dialysis; Tac: tacrolimus.

**Table S2:** Baseline demographic, clinical, and laboratory characteristics of unmatched patients in both groups.

Characteristics	Non-CAKUT (n = 151)	CAKUT (n = 6)
Donor age (years), median (IQR)	46.5 (34–53)	42.5 (32.5–48.8)
Donor sex, %		
Male	56.3	50
Female	43.7	50
Recipient sex, %		
Male	57.6	66.7
Female	42.4	33.3
Recipient age (years), median (IQR)	34 (26–45)	30.5 (20.8–35)
Donor type, %		
Living	76.1	83.3
Deceased	23.8	16.6
Follow-up duration (months), median (IQR)	110 (80–159)	29.5 (20.5–61.5)
HLA mismatches, %		
≤3	70.8	100
>3	29.1	0
Duration of dialysis (months), median (IQR)	20 (6–48)	207 (180–234)
Previous transplantation, %	0.06	16.6
PRA ≥30% before transplantation, %	0.06	0
Pretransplant KRT, %		
No (Preemptive)	15.9	0
Yes (HD and/or PD)	84.1	100
Induction, %		
No	55.6	66.7
Basiliximab	19.9	16.6
ATG	24.5	16.6
Immunosuppressive regimen, %		
Tac/MPA/steroids	43.7	66.7
CsA/MPA/steroids	19.2	0
Tac/AZA/steroids	8.6	16.6
CsA/AZA/steroids	2.6	0
Others	25.8	16.6

**Abbreviations:** ATG: anti-thymocyte globulin; AZA: azathioprine; CAKUT: congenital anomalies of the kidney and urinary tract; CsA: cyclosporine; HD: hemodialysis; HLA: human leukocyte antigen; IQR: interquartile range; KRT: kidney replacement therapy; MPA: mycophenolic acid; PD: peritoneal dialysis; Tac: tacrolimus.

## Figures and Tables

**Table 1:** Baseline demographic, clinical, and laboratory characteristics of all patients.

Characteristics	Non-CAKUT (n = 169)	CAKUT (n = 169)	p
Donor age (years), median (IQR)	45.0 (35.0–56.0)	45.5 (39.0–57.0)	0.36
Donor sex, n (%)			
Male	46.2	43.2	0.58
Female	53.8	56.8	
Recipient sex, n (%)			
Male	64.5	54.4	0.06
Female	35.5	45.6	
Recipient age (years), median (IQR)	30 (25.0–38.0)	25.5 (20.0–30.0)	0.001
Discharge creatinine (mg/dL), median (IQR)	1.0 (0.8–1.2)	1.0 (0.8–1.0)	0.126
Discharge GFR (mL/min/1.73 m^2^), median (IQR)	89.0 (68.5–109.2)	86.0 (66.0, 106.5)	0.331
Donor type, %			
Living	73.4	73.4	1
Deceased	26.6	26.6	
Follow-up duration (months), median (IQR)	133 (75–179)	139.5 (121.5–202.3)	0.85
HLA mismatches, %			0.49
≤3	17.8	20.7	
>3	82.2	79.3	
Duration of dialysis (months), median (IQR)	15 (5–30)	19 (6.5–43)	<0.001
Previous transplantation, %	4.1	0	0.008
PRA ≥30% before transplantation, %	1.1	4.7	0.054
Pretransplant KRT, %			
No (Preemptive)	9.5	10.1	0.86
Yes (HD and/or PD)	90.5	89.9	
Induction, %			0.418
No	57.9	61.5	
Basiliximab	14.8	10	
ATG	27.2	28.4	
Immunosuppressive regimen, %			0.28
Tac/MPA/steroids	53.4	45.6	
CsA/MPA/steroids	22.1	18.4	
Tac/AZA/steroids	8.4	9.5	
CsA/AZA/steroids	3.1	6.1	
Others	13.0	20.4	

**Abbreviations:** ATG: antithymocyte globulin; AZA: azathioprine; CAKUT: congenital anomalies of the kidney and urinary tract; CsA: cyclosporine; GFR: glomerular filtration rate; HD: hemodialysis; HLA: human leukocyte antigen; IQR: interquartile range; KRT: kidney replacement therapy; MPA: mycophenolic acid; PD: peritoneal dialysis; Tac: tacrolimus.

**Table 2:** Posttransplant outcomes of study and control groups.

Outcomes	Non-CAKUT (n = 169)	CAKUT (n = 169)	p
Biopsy-proven rejection, %			
Acute TCMR	10.1	7.1	0.27
Acute ABMR	0.6	3.6	
Chronic ABMR	7.7	8.9	
Chronic TCMR	1.2	0.0	
Borderline	0.6	1.8	
Mixed	0.6	0.6	
ABMR and TCMR in different times	0.6	0.6	
Urinary tract infections, %	10.7	20.7	0.01
Proteinuria (g/g) at last visit, median (IQR)	1.3 (0.1–3)	0.33 (0.1–1.2)	<0.001
eGFR (mL/min/1.73 m^2^) at last visit, median (IQR)	32.3 (11.0–67.3)	53.7 (23.9–77.2)	<0.001
Medication nonadherence, %	3.0	9.5	0.01
Posttransplant DSA development, %	12.7	10.7	0.59
BK nephropathy, %	0.0	1.8	0.08
CNI toxicity, %	16.0	9.5	0.054
Chronic allograft nephropathy, %	14.8	14.8	0.12
Graft loss, %	49.7	23.7	<0.001
Chronic allograft nephropathy	7.7	7.1	
Rejection	15.5	11.2	
Recurrent/de novo glomerulonephritis	21.4	0.6	
BK nephropathy	0.0	0.6	
Thrombotic microangiopathy	0.6	0.0	
Death with a functioning graft	4.7	3.6	
Sepsis induced	0.0	0.6	
Death^*^, %	11.2	5.3	<0.001

**Abbreviations:** CAKUT: congenital anomalies of the kidney and urinary tract; IQR: interquartile range; TCMR: T cellmediated rejection; ABMR: antibody-mediated rejection; DSA: donor specific antibody; CNI: calcineurin inhibitors; eGFR: estimated glomerular filtration rate.

**Table 3:** Various clinical features and outcomes and of patients with VUR nephropathy and other patients with CAKUT.

Characteristics	Other patients with CAKUT (n = 40)	VUR nephropathy (n = 129)	p
Pretransplant PRA, %	5	1.5	0.60
Posttransplant PRA development, %	25	15.0	0.14
Posttransplant DSA development, %	18	8.5	0.10
BPR, %	25	15.5	0.17
eGFR (mL/min/1.73 m^2^) at last visit, median (IQR)	80.2 (38.9–101.3)	57.4 (30–85.3)	0.02
Proteinuria (g/g) at last visit, median (IQR)	0.2 (0.1–0.8)	0.2 (0.1–0.8)	0.96
Urinary tract infections, %	7.5	24.8	0.02
Problems of medication adherence, %	10	9.3	0.89
Posttransplant VUR, %	2.5	12.4	0.07
BK nephropathy, %	5	0.8	0.08
CNI toxicity, %	10	9.3	0.88
Chronic allograft nephropathy, %	10	16.3	0.43
Graft loss, %	20	24.8	0.53
Death^*^, %	5	5.4	0.92

**Abbreviations:** CAKUT: congenital anomalies of the kidney and urinary tract; IQR: interquartile range; PRA: panel reactive antibody; BPR: Biopsy-proven acute rejection; VUR: vesicoureteral reflux; DSA: donor specific antibody; CNI: Calcineurin inhibitors; eGFR: estimated glomerular filtration rate.
